# Dynamics of Transcription Factors in Three Early Phases of Osteogenic, Adipogenic, and Chondrogenic Differentiation Determining the Fate of Bone Marrow Mesenchymal Stem Cells in Rats

**DOI:** 10.3389/fcell.2021.768316

**Published:** 2021-10-26

**Authors:** Qingyu Zhang, Jun Dong, Peng Zhang, Dongsheng Zhou, Fanxiao Liu

**Affiliations:** Department of Orthopaedics, Shandong Provincial Hospital Affiliated to Shandong First Medical University, Jinan, China

**Keywords:** bone marrow mesenchymal stem cells (BMSCs), high-throughput sequencing, osteogenic differentiation, chondrogenic differentiation, transcription factors, adipogenic differentiation

## Abstract

The imbalance of osteogenic, adipogenic, and chondrogenic differentiation in bone marrow mesenchymal stem cells (BMSCs) occurred in multiple age-related degenerative diseases such as osteoporosis and osteoarthritis. In order to improve our understanding and control of multi-directional differentiation of BMSCs in rats, using high-throughput sequencing, we identified key gene regulatory events in the early stages of lineage commitment. Data analysis revealed two transcription factors (TFs, Tsc22d3, and Epas1) with elevated expression throughout the initiation of differentiation (3 h), lineage acquisition (12 h), and early lineage progression (72 h) of three-directional differentiation. For osteogenic differentiation, 792, 1,042, and 638 differentially expressed genes including 48, 59, and 34 TFs were identified at three time points, respectively. Moreover, the functional analysis demonstrated that 4, 12, and 5 TFs were only differentially expressed during osteogenic differentiation at 3, 12, and 72 h, respectively, and not during other two-directional differentiation. Hopx showed enhanced expression throughout three early phases during the osteogenic differentiation but no significant change in other two-directional differentiation. A similar pattern of Gbx2 expression occurred in chondrogenic differentiation. Thus, Hopx and other early responder TFs may control the osteogenic cell fate of BMSCs and participate in the development of osteoporosis. Gbx2 and other early responder TFs should be considered in mechanistic models that clarify cartilage-anabolic changes in the clinical progression of osteoarthritis.

## Introduction

Bone mesenchymal stem/stromal cells (BMSCs) have the potentials of differentiating into several mesodermal derivatives, in particular osteogenic (osteogenic progenitors, osteocytes, osteoblasts, and bone lining cells), adipogenic and chondrogenic cells in the existence of physiological demands or pathological changes (Arthur and Gronthos, 2020; Jiang et al., 2021). Systematic cell-based therapy by administration of *in vivo* expanded BMSCs could serve as an excellent alternative for bone regeneration and tissue engineering (Arthur and Gronthos, 2020; Robert et al., 2020; Jiang et al., 2021), but it is not yet a standard clinical practice due to donor variation among patients and unpredictable direction for cell differentiation (Crapnell et al., 2013; O’Connor, 2019).

The adipogenesis and osteogenesis of BMSCs are competing and reciprocal, keeping homeostasis within the bone. The primary role of BMSCs-derived osteoblasts is to produce and secrete osteoid and mineralizing factors to remodel bone according to the metabolic and structural needs of the body (Yu and Wang, 2016). During aging and the development of bone remodeling disorders such as osteoporosis and osteonecrosis, the normal balance between osteogenic and adipogenic cell populations can shift toward the latter and result in increased formation of marrow fat (Zhang et al., 2018; Pittenger et al., 2019; Robert et al., 2020; Saeedi et al., 2021). Hyaline cartilage is responsible for bone formation in the embryo and can be found in the articular surface of the bones of adults (Carballo et al., 2017). Until now, the *in vitro* differentiation process is unable to produce cartilage similar to articular cartilage formed under normal physiological conditions (Huynh et al., 2019). Further understanding the mechanism for the three-directional differentiation of BMSCs is imperative.

The differentiation of BMSCs is a two-step process, lineage commitment (from MSCs to lineage-specific progenitors) and maturation (from progenitors to specific cell types) (Robert et al., 2020). The lineage commitment of MSCs to adipocytes, osteoblasts and chondrocytes share a common precursor (Chen et al., 2016; Yianni and Sharpe, 2020), and intensive studies in recent decades have demonstrated that a number of signaling pathways and transcription factors (TFs) are critical for this process (van de Peppel et al., 2017). RUNX2, SP7/Osterix, and SOX9 are among the leading TFs involved in the differentiation of MSCs into osteoblasts or chondrocytes, while PPARγ and cyclic AMP response element-binding protein (CREB) are required for adipogenic differentiation (Chen et al., 2016; Pittenger et al., 2019; Robert et al., 2020). Overexpression of RUNX2 in non-osteoblastic cells and adipose-tissue-derived MSCs elevates the level of osteoblastic markers, promotes mineralization and inhibits lineage commitment to adipocytes, indicating that a single TF is important for bone development and osteoblast differentiation (Robert et al., 2020). During adipogenesis, the production of cyclic AMP is augmented, resulting in phosphorylation of CREB and an increased expression of CEBPβ (Merrett et al., 2020). CEBPβ, together with the other two CEBP family members (CEBPα and CEBPδ), was reported to be implicated in adipogenic differentiation (Chen et al., 2016; Hu et al., 2018; Robert et al., 2020).

Although the enriched biological processes and TFs are mostly identical between these lineages within the first 4 days after induction (van de Peppel et al., 2017; Fang et al., 2020), the early stages of lineage commitment of human BMSCs could be further divided into three distinct phases with own mRNA dynamics: initiation of differentiation (0–3 h, phase I), lineage acquisition (6–24 h, phase II), and early lineage progression (48–96 h, phase III) (van de Peppel et al., 2017). Downstream analyses of four TFs (TP53, FOSl1, ESR1, and HOXA9) identified during the first phase of osteogenic differentiation are capable of regulating more than 50% of the differentially expressed genes (DEGs) in the second and third phases. However, the expression of TFs in chondrogenic differentiation during this stage was not systematically investigated. Meanwhile, this study used human BMSCs, while key TFs playing critical roles in lineage commitment of rat BMSCs were not considered.

BMSCs from 4 different species (human, pig, rat, guinea pig) fail to elicit a proliferative response from allogeneic lymphocytes and therefore can function across the species barrier (Li et al., 2012). Given the unique immunologic properties of rat BMSC, BMSC from rats is an attractive option for xenografts. In the current study, *in vivo* differentiation of rat BMSCs from a two-dimensional culture system was applied, and by using high-throughput transcriptome sequencing technique and multiple bioinformatics methods, we systematically investigated genes, especially TFs being regulated upon lineage commitment of rat BMSCs into adipocytes, chondrocytes, and osteocytes. One of our key findings was the characterization of genes and regulatory programs controlling the early stages of lineage commitment of rat BMSCs. Progress in this area will be of great value to understand age- and pathology-related fractures and metabolic syndromes, and to guide a better application of BMSCs in tissue engineering and regenerative medicine.

## Materials and Methods

### Cell Isolation and Induced Differentiation

This study was approved by the Animal Ethics Committee of Shandong Provincial Hospital affiliated to Shandong First Medical University. BMSCs were collected from femurs and tibia of 3 female Sprague Dawley (SD) rats (3 weeks old) by washing the marrow cavity using high glucose Dulbecco’s Modified Eagle Medium (DMEM) (Gibco, Rockville, MD, United States) until bones appear white. Obtained cell clumps were dispersed to cell suspension by using 21-gauge needles. The cells were cultured in DMEM supplemented with 10% fetal bovine serum (FBS) in an incubator of 5% CO_2_ and 95% humidity at 37°C. BMSCs at passage 2 were seeded in 12-well cell culture plates and after 2 days, cells were induced to differentiate into osteoblasts using mesenchymal stem cell osteogenic differentiation medium (Promocell, Germany, C-28013), or to adipocytes using mesenchymal stem cell adipogenic differentiation medium (Promocell, Germany, C-28016), or to chondrocytes using mesenchymal stem cell chondrogenic differentiation medium (Promocell, C-28012). Differentiation media were replaced every 3–4 day.

### Protein Concentration Assay and Bone Marrow Mesenchymal Stem Cells Staining

Protein concentration assay, Elisa assay, alizarin red staining, Oil Red O staining and Alcian blue staining were performed as previously described (Bruedigam et al., 2011; Gari et al., 2016).

### RNA Isolation

To obtain enough RNA for the gene expression profiling analyses, we pooled three individual cultures in TRIzol (Life Technologies), resulting in a total of three experimental samples per time point (3 time points, namely, 3, 12, and 72 h) per lineage (osteogenic, adipogenic, or chondrogenic) to be used for gene expression profiling analyses. Plus, the control samples, there were a total of 30 samples. RNA was isolated as previously described (Bruedigam et al., 2011).

### High-Throughput Sequencing

The quality of RNA was analyzed using NanoDrop^TM^ One (Thermo Fisher Scientific, United States). An mRNA library was constructed by NEBNext^®^ Ultra^TM^ II mRNA Library Prep Kit for Illumina^®^ (NEB, United States). Qubit^TM^ dsDNA HS Assay Kit (Invitrogen, United States), D1000 Screen Tape (Agilent, United States), and KAPA Library Quant kit (illumine, United States) universal qPCR Mix were used to assess the quality and yield of the constructed library. Finally, the mRNA was sequenced by NovaSeq 6000 (Illumina, United States).

### Sequencing Result Analysis

After standardization and quality control of the sequencing data, the differential expression of mRNA was analyzed. | log2fold change (FC)| in mRNA expression ≥ 1 with an adjusted *p*-value < 0.05 was used as the threshold for determining gene upregulation or downregulation. Differentially expressed TFs were identified by using the AnimalTFDB 3.0 database.^1^ Gene Ontology (GO) enrichment^2^ and Kyoto Encyclopedia of Genes and Genomes (KEGG) enrichment^3^ analysis was utilized to determine the biological function of the differentially expressed TFs and investigate its possible involvement in the lineage-commitment of rat BMSCs. The protein-protein interaction (PPI) network of identified genes was predicted using the multiple protein online tool in STRING database (Liu et al., 2021), a search tool for the Retrieval of Interacting Genes database (version 11.0b)^4^ and visualized in Cytoscape software (Version 3.6.2). Relationships between critical TFs and their differentially expressed target genes were predicted to construct the TF-target regulatory network based on the previous method (Zhang et al., 2017; Zhou et al., 2021).

### Statistical Analysis

Statistical analyses were conducted by using SPSS Software, version 17.0 (IBM SPSS Statistics, United States). One-way analysis of variance (ANOVA) followed by LSD method was used to compare the protein concentration and ALP expression between different groups, which are expressed as the means ± standard deviation. Differences between means were considered statistically significant when *p*-values were < 0.05.

## Results

### Differentiation of Rat Bone Marrow Mesenchymal Stem Cells Into Osteoblasts, Adipocytes, and Chondrocytes

**FIGURE 1:**
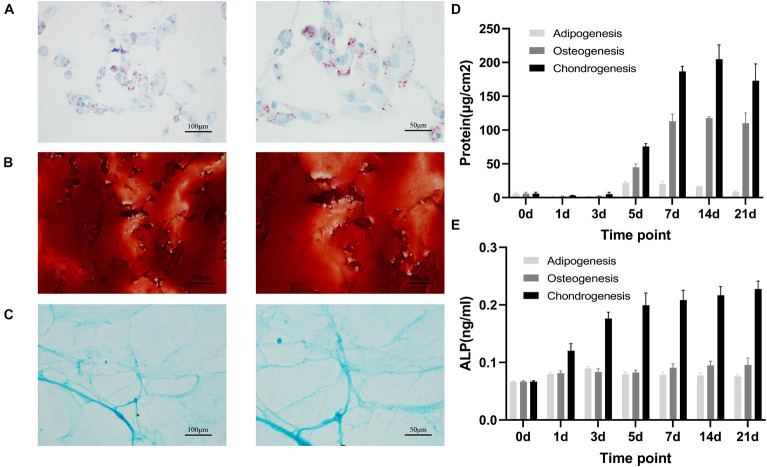
Adipogenic, osteogenic and chondrogenic differentiation of rat BMSCs. Histochemical staining of calcium **(A)** with Alizarin red or adipocyte **(B)** with oil red O or cartilage **(C)** with Alcian blue staining after 21 days of osteogenic or adipogenic or chondrogenic differentiation. Scale bars, 100 and 50 μm; total protein **(D)** and ALP **(E)** concentration in osteogenesis, adipogenesis and chondrogenesis of rat BMSC. Error bars indicate SD. Mean and SD of three independent experiments with three technical replicates.

On day 21 after induced differentiation, histological staining for calcium in the osteogenic differentiation group showed that extracellular matrix (ECM) was mineralized (Figure 1A), Oil Red O staining for lipids in the adipogenic differentiation group showed that cells accumulated intracellular lipid vesicle (Figure 1B), and Alcian blue staining in the chondrocyte differentiation group showed cartilage formation (Figure 1C). Biochemical analyses of samples during differentiation revealed that total protein and alkaline phosphatase (ALP) concentration transiently increased during adipogenic, osteogenic, and chondrogenic differentiation (Figures 1D,E). Together, our observations established that rat BMSCs differentiated into three lineages, consistent with their expected multi-lineage potential.

### Identification of Differentially Expressed Transcription Factors in Three Phases

**FIGURE 2:**
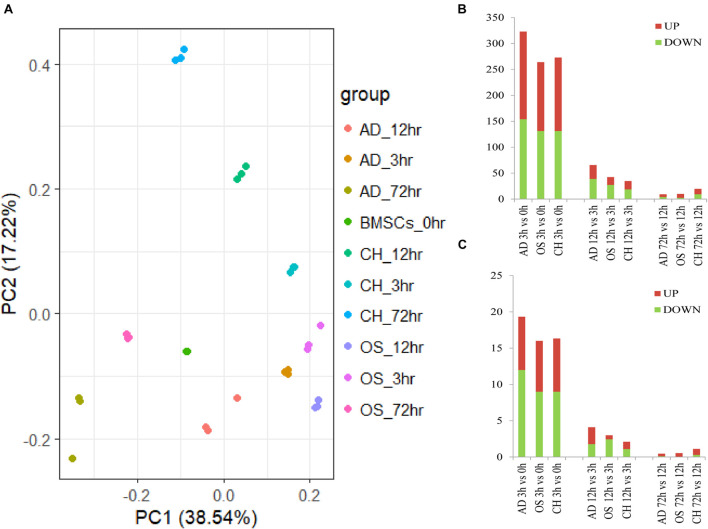
Dynamic transcriptional changes upon osteogenic, adipogenic, and chondrogenic differentiation of rat BMSCs. **(A)** Principal component analyses of the gene expression profile in osteogenic, adipogenic, and chondrogenic groups at three time points; **(B)** number of significant differential TFs relative to the previous time point (based on three independent experiments); and **(C)** the number of differentially expressed TFs per hour compared to the previous time point (based on three independent experiments). AD, adipogenesis; OS, osteogenesis; CH, chondrogenesis.

Data cross−comparability was evaluated via principal component analysis (PCA) for confirming biological variability between different samples. All samples were grouped separately (Figure 2A), indicating globally distinct expression profiles. Analysis of gene expression dynamics during rat BMSC differentiation revealed that transcript levels changed significantly 3 h upon induction of differentiation (Figure 2B). During induction of osteogenic differentiation, 792, 1,042, and 638 DEGs were identified at three time points, respectively. At least 48 TFs (21 upregulated and 27 downregulated ones) were significantly different at 3 h and this increased further to 59 (25 upregulated and 34 downregulated ones) at 12 h. The number of significantly modulated TFs decreased to 34 (22 upregulated and 12 downregulated ones) at 72 h. During adipogenic differentiation, the number of differentially expressed TFs was 58 at 3 h, 26 at 12 h, and 36 at 72 h. During chondrogenic differentiation, the number was 49 at 3 h, 38 at 12 h, and 39 at 72 h. Remarkably, at 3 and 12h after differentiation, the number of downregulated TFs was higher than the upregulated ones. Next, we calculated the number of differentially expressed TFs at each time point compared with the preceding time point and in three directions of differentiation, the number of TFs that changed per hour decreased as time extends (Figure 2C). Differentially expressed TFs in three time points during three-directional differentiation were summarized in Table 1.

**TABLE 1:** The same differential expressed TFs at three time points after differentiation into osteoblasts, adipocytes, and chondroblasts.

Groups	Total	TFs	Groups	Total	TFs
OS3h, 12h, 72h; AD3h, 12h, 72h, CH3h, 12h, 72h	2	Tsc22d3, Epas1	CH3h, 12h, 72h; OS72h	1	Plscr1
OS3h, 12h, 72h; AD3h, 12h, 72h, CH3h, 72h	2	Rab24, Cenpa	CH3h, 12h; OS3h, 12h	1	Atoh8
OS12h; AD3h, 12h, 72h; CH3h, 12h, 72h	1	Sox9	AD3h, 12h; CH12h	1	Klf5
OS3h, 12h; AD3h, 12h, 72h, CH3h, 12h	2	Id3, Id2	AD3h; CH3h; OS3h	1	Hmg20a
OS72h; AD3h, 12h, 72h; CH12h, 72h	1	Snai2	AD3h; OS3h, 12h	2	Phb, Emx2
OS3h, 12h; AD3h, 12h; CH3h, 72h	1	Ahrr	AD12h; CH72h; OS12h	1	Bhlhe41
OS12h, 72h; AD3h, 72h; CH12h, 72h	1	Klf10	AD72h; CH72h; OS72h	1	Tfcp2l1
OS3h, 12h, 72h; AD3h; CH3h, 12h	1	Meox2	CH3h, 12h, 72h	1	Gbx2
OS72h; AD12h, 72h; CH3h, 12h, 72h	1	Hic1	CH3h, 12h; OS3h	2	Sox11, AABR07001099.1
AD3h, 12h, 72h; CH3h, 72h	1	Irf5	CH3h, 12h; OS12h	1	Tbx4
AD3h, 12h, 72h; CH12h, 72h	1	Bhlhe40	CH3h; OS3h, 12h	1	Hoxd13
OS3h, 12h; AD3h, 12h; CH3h	2	Cenpt, Foxm1	CH12h; OS3h, 12h	1	Nme2
OS3h, 12h, 72h; AD3h, 72h	1	Klf4	CH12h; OS12h, 72h	1	AABR07015743.1
OS3h, 12h; AD3h; CH3h, 12h	1	Hbp1	OS3h, 12h, 72h	1	Hopx
OS3h, 12h; AD3h; CH3h, 72h	1	Zfp467	AD3h; CH3h	2	Zfp668, Mef2a
OS3h, 12h, 72h; AD3h; CH72h	1	Dlx3	AD3h; CH72h	1	Ets2
OS3h, 12h; AD12h, 72h; CH72h	1	Ehf	AD3h; OS3h	1	Foxf1
OS72h; AD72h; CH3h, 12h, 72h	2	Nfia, Scx	AD72h; CH12h	1	Nr1d1
OS12h, 72h; AD72h; CH3h, 72h	1	Pax8	AD72h; OS72h	2	Ascl2, Zfp503
OS3h, 12h, 72h; AD72h; CH3h	1	Mafb	OS12h; CH3h	1	Hey2
OS3h, 12h; CH3h, 12h, 72h	1	Tshz2	AD3h, 12h	2	Zfp667, Nr4a1
OS12h, 72h; CH3h, 12h, 72h	1	Tsc22d1	AD12h, 72h	1	Arid3b
OS12h; AD3h, 12h, 72h	1	Hoxd9	CH3h, 12h	2	Zfp394, Jun
AD3h, 12h, 72h; OS72h	1	Zfp536	CH12h, 72h	2	Gsc, Terf1
AD3h, 12h; CH3h; OS12h	1	Relb	OS3h, 12h	4	Zfp773-ps1, Klf16, Zfp367, Foxn1
AD3h, 72h; CH3h; OS3h	1	Klf3	OS3h, 72h	1	Zfp296
AD3h, 72h; OS3h, 12h	1	Zfp521	OS12h, 72h	2	Rfx2, Hif1a
AD3h; CH3h, 12h; OS3h	1	Hoxc6	OS3h	4	Ybx3, Zbtb25, Hes6, Zfp879
AD3h; CH3h, 72h; OS12h	1	Rfx3	OS12h	12	Gtf3a, Hoxd3, LOC500584, Csdc2, Maf, Zfp113, Zfp174, LOC108349189, E2f2, Rbpjl, Pou5f2, AABR07029613.1
AD3h; CH3h; OS3h, 12h	3	Tbx6, Foxc2, Mxd4	OS72h	5	Tmf1, Msx2, Tcf23, Rfx8, Pparg
AD3h; CH12h; OS3h, 72h	1	Zfp39	AD3h	15	Pou6f1, Sp5, L3mbtl1, Lhx9, Zbtb38, Hoxd10, Pax4, Arid2, Zfp317, Zeb1, Tulp1, Zfp541, Pou2f3, Foxc1, Zbtb12
AD3h; OS3h, 12h, 72h	1	Tead3	AD12h	1	Atf4
AD12h, 72h; CH12h, 72h	1	Creb3l1	AD72h	5	RGD1560108, Hlx, Foxo3, Osr1, LOC100362054
AD12h; CH3h, 72h; OS12h	1	Hmgb2	CH3h	7	Preb, Csrnp3, Mafa, Lef1, Stat6, Prdm16, Zfp395
AD72h; OS3h, 12h, 72h	1	Zfhx2	CH12h	4	Lrrfip2, Zfp62, Tfam, AABR07060287.1
CH3h, 12h, 72h; OS3h	1	Runx3	CH72h	8	Smarca1, Myrfl, Mlxip, Dlx5, Rarb, Heyl, Foxd2, Gli1

*OS, osteogenic differentiation; CH, chondrogenic differentiation AD, adipogenic differentiation; TF, transcription factor.*

**FIGURE 3:**
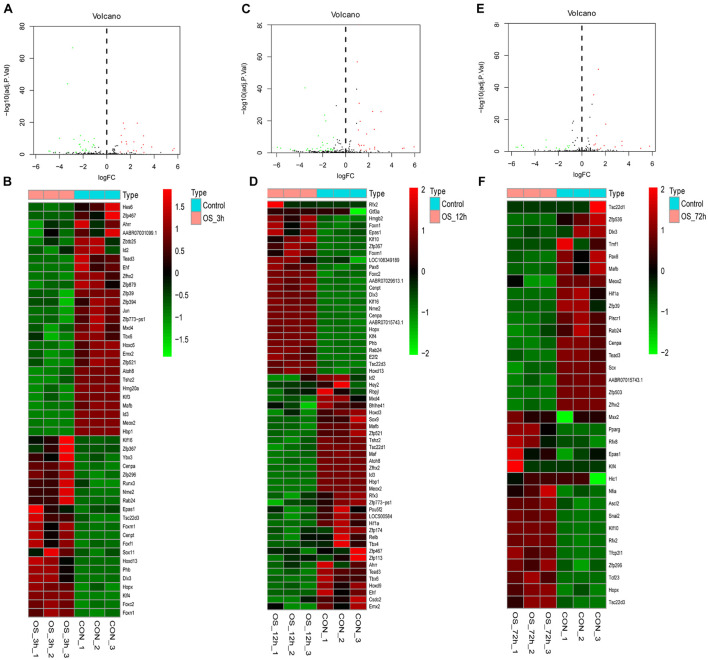
Volcano plot and heatmap of differentially regulated TFs during osteogenesis at 3 h **(A,B)**, 12 h **(C,D)**, and 72 h **(E,F)** compared with undifferentiated cells (*t* = 0 h).

**FIGURE 4:**
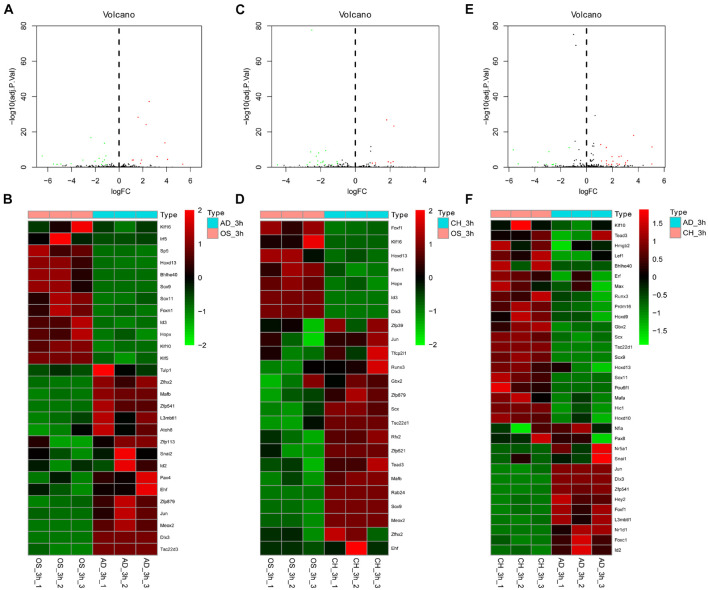
Volcano plot and heatmap of differentially regulated TFs between every two lineages at 3 h after inductive differentiation (*t* = 0 h). **(A,B)** comparison between osteogenesis and adipogenesis; **(C,D)** comparison between osteogenesis and chondrogenesis, and **(E,F)** comparison between chondrogenesis and adipogenesis. AD, adipogenesis; OS, osteogenesis; CH, chondrogenesis.

The volcano plot and heatmap of differentially expressed TFs at 3, 12, and 72 h of osteogenesis were shown in Figure 3, while results of comparison between every two lineage-commitment at 3 h after inductive differentiation were presented in Figure 4. Supplementary Figures 1, 2 described the visualized results of differentially expressed TFs at three time points of adipogenic and chondrogenic differentiation.

### Identification of Specific Differentially Expressed Transcription Factors in Three Phases

**FIGURE 5:**
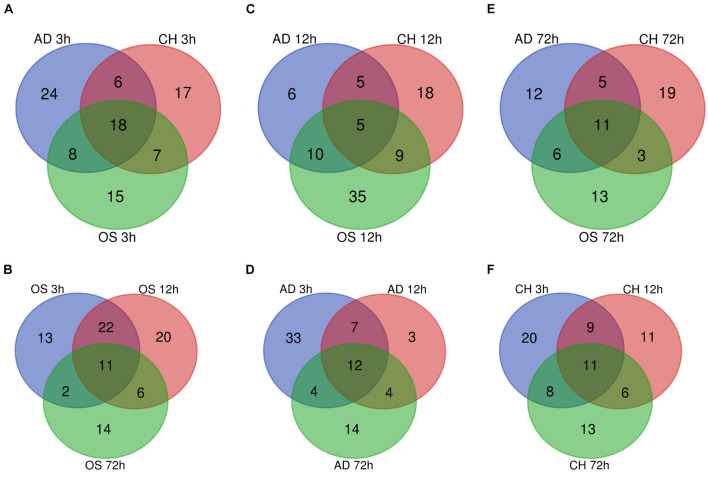
Venn Diagrams of differentially regulated TFs at 3 h **(A)**, 12 h **(C)**, and 72 h **(E)** of three directions of differentiation compared with undifferentiated cells (t = 0 h). Venn Diagrams of differentially regulated TFs during osteogenesis **(B)**, adipogenesis **(D)**, and chondrogenesis **(F)** at three time points compared with undifferentiated cells (t = 0 h). AD, adipogenesis; OS, osteogenesis; CH, chondrogenesis.

In osteogenesis of BMSCs, there were 13, 20, and 14 TFs being identified to be differentially expressed only at 3, 12, and 72 h after inductive differentiation (Figure 5A). In adipogenesis, the numbers were 33, 3, and 14 (Figure 5B), and for chondrogenesis, the numbers were 20, 11, and 13 (Figure 5C). Notably, 11 (e.g., Hopx and Tsc22d3), 12 (e.g., Irf5 and Rab24), and 11 (e.g., Gbx2 and Epas1) TFs showed differentially expressed levels at all three time points in osteogenesis, adipogenesis, and chondrogenesis, respectively.

### Comparison of Differentially Expressed Transcription Factors Between Different Lineages

At 3 h after induction of differentiation, it was noticed that there were 15, 24, and 17 TFs only differentially expressed in osteogenesis, adipogenesis, and chondrogenesis, respectively (Figure 5D). At 12 h, the numbers were 35, 6, and 18 (Figure 5E), and at 72 h, the numbers were 13, 12, and 19 (Figure 5F). Notably, 11 (e.g., Hmg20a and Hoxc6), 5 (e.g., Id2 and Id3), and 11(e.g., Tfcp2l1 and Scx) TFs showed differential expression at all three directions of differentiation at 3, 12, and 72 h after inductive differentiation, respectively.

### Functional Enrichment Analysis of Differentially Expressed Transcription Factors

**FIGURE 6:**
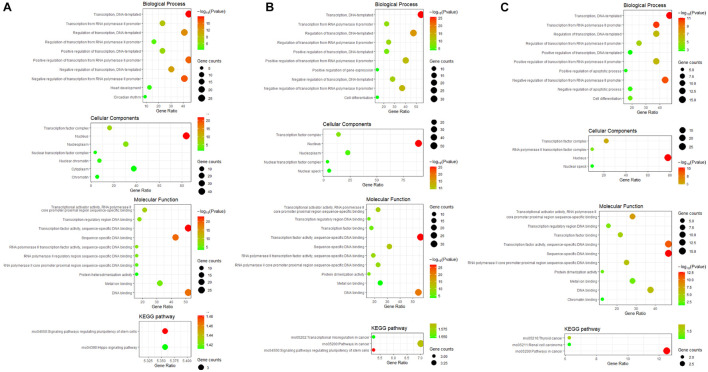
GO including biological process, cellular components and molecular function analysis, and KEGG enrichment analysis of differentially expressed TFs at 3 h **(A)**, 12 h **(B)**, and 72 h **(C)** of osteogenesis compared with undifferentiated cells (t = 0 h).

GO and KEGG pathway enrichment analysis of differentially expressed TFs at different time points of three directions of differentiation was performed to identify the most relevant biological processes (BPs), molecular functions (MFs), cellular components (CCs), and pathways. The top enriched terms in BP, CC, MF, and KEGG at 3 h after osteogenesis were presented in Figure 6A. For instance, the enriched BPs terms included “negative regulation of transcription from RNA polymerase II promoter,” “regulation of transcription, DNA-templated,” and “positive regulation of transcription from RNA polymerase II promoter.” The enriched terms at 12 and 72 h after osteogenesis were presented in Figures 6B,C, respectively, while results of the other two directions of differentiation were presented in Supplementary Figures 3, 4.

**FIGURE 7:**
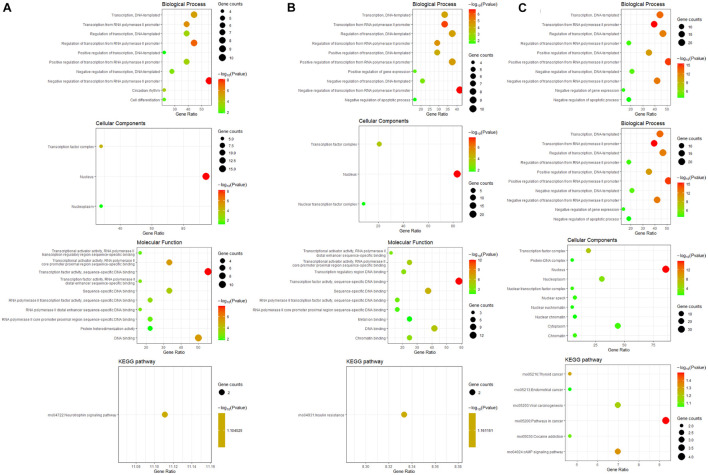
GO including biological process, cellular components and molecular function analysis, and KEGG enrichment analysis of differentially expressed TFs between every two lineages at 3 h after inductive differentiation (*t* = 0 h). **(A)** comparison between osteogenesis and adipogenesis; **(B)** comparison between osteogenesis and chondrogenesis, and **(C)** comparison between chondrogenesis and adipogenesis.

The differentially expressed TFs between osteogenesis and adipogenesis at 3 h after inductive differentiation were also significantly enriched in BPs terms such as “negative regulation of transcription from RNA polymerase II promoter,” “regulation of transcription, DNA-templated.” Results of functional enrichment analysis of the differentially expressed TFs between every two lineage-commitment at 3 h of inductive differentiation were presented in Figure 7.

### Protein Interaction Network Construction of Differentially Expressed Transcription Factors

**FIGURE 8:**
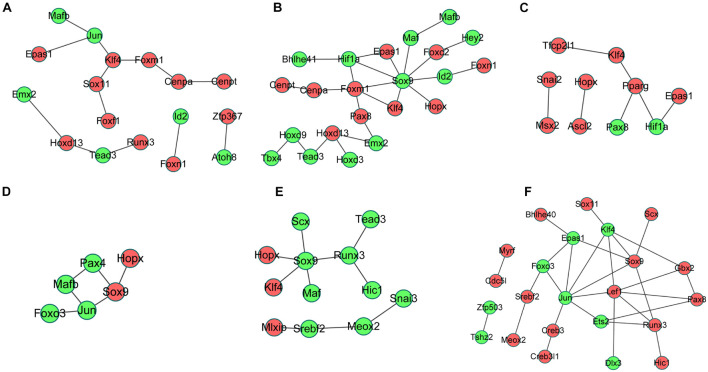
Protein-protein interaction (PPI) network analysis of differentially expressed TFs. PPI network of differentially expressed TFs at 3 h **(A)**, 12 h **(B)**, and 72 h **(C)** of osteogenesis compared with undifferentiated cells (*t* = 0 h); the PPI network of differentially regulated TFs between osteogenesis and adipogenesis **(D)**, between osteogenesis and chondrogenesis **(E)**, and between chondrogenesis and adipogenesis **(F)** at 3 h after inductive differentiation (*t* = 0 h). Red indicates upregulated TFs, and green indicates the downregulated TFs.

The interactions between the proteins expressed from differentially expressed TFs at 3 h of osteogenesis consisted of 17 nodes and 13 edges, with Klf4 gene being identified to have a relatively high connectivity degree (Figure 8A). The PPI networks at 12 and 72 h after osteogenesis were presented in Figures 8B,C, respectively. Meanwhile, the PPI networks of the other two-direction differentiation at three time points were presented in Supplementary Figure 5.

The interactions between the proteins expressed from differentially expressed TFs between osteogenesis and adipogenesis at 3 h after inductive differentiation consisted of 6 nodes and 6 edges, with the Sox9 gene being identified to have a relatively high connectivity degree (Figure 8D). The interactions between the proteins expressed from differentially expressed TFs between osteogenesis and chondrogenesis, and between adipogenesis and chondrogenesis at 3 h after inductive differentiation were presented in Figures 8E,F.

### Construction of Differentially Expressed Transcription Factors and Differentially Expressed Genes Regulatory Network

**FIGURE 9:**
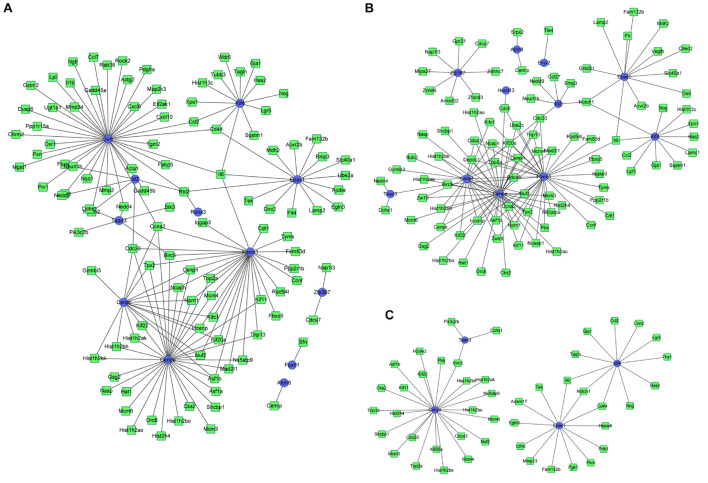
The regulatory network between differentially expressed TFs at 3 h and targeted differentially expressed genes at 3 h **(A)**, 12 h **(B)**, and 72 h **(C)** in osteogenesis compared with undifferentiated cells (*t* = 0 h). Blue indicates TFs, and green indicates targeted genes.

**FIGURE 10:**
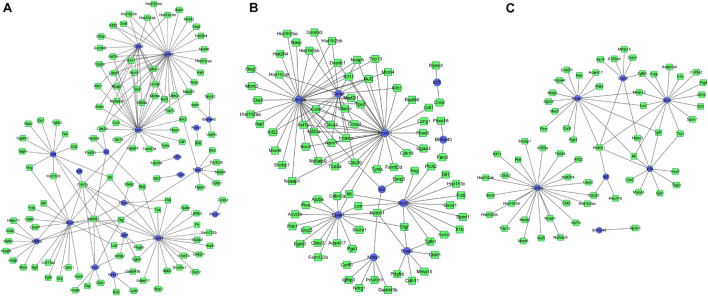
The regulatory network between differentially expressed TFs at 3 h and targeted differentially expressed genes at 3 h **(A)**, 12 h **(B)**, and 72 h **(C)** in adipogenesis compared with undifferentiated cells (*t* = 0 h). Blue indicates TFs, and green indicates targeted genes.

**FIGURE 11:**
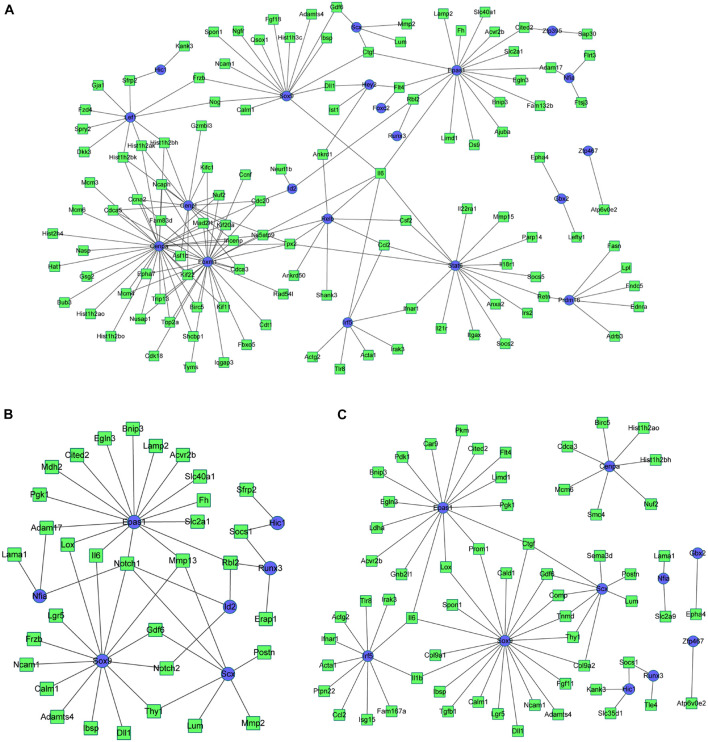
The regulatory network between differentially expressed TFs at 3 h and targeted differentially expressed genes at 3 h **(A)**, 12 h **(B)**, and 72 h **(C)** in chondrogenesis compared with undifferentiated cells (*t* = 0 h). Blue indicates TFs, and green indicates targeted genes.

Based on the integrated analysis between TFs and DEGs, it could be noticed that during osteogenesis, the differentially expressed TFs at 3 h were able to regulate 111, 92, and 46 DEGs at 3, 12, and 72 h after differentiation (Figures 9A–C). Four differentially expressed TFs (Tead3, Cenpa, Epas1, and Klf4) identified at 3 h could function at all three time points. Meanwhile, during adipogenesis, the differentially expressed TFs at 3 h were able to regulate 113, 85, and 61 DEGs at 3, 12, and 72 h after differentiation (Figures 10A–C). Six differentially expressed TFs (Sox9, Snai2, Epas1, Id2, Cenpa, and Bhlhe40) occurred in three regulatory networks. For chondrogenesis, the differentially expressed TFs at 3 h were able to regulate 115, 33, and 60 DEGs at 3, 12, and 72 h after differentiation (Figures 11A–C). Six differentially expressed TFs (Runx3, Hic1, Scx, Sox9, Epas1, and Nfia) identified at 3 h could function at all three time points.

The regulatory networks between TFs and DEGs at 12 and 72 h after differentiation were presented in Supplementary Figure 6. The regulatory networks of the differentially expressed TFs and DEGs between every two lineage-commitment at 3 h after inductive differentiation were presented in Supplementary Figure 7.

## Discussion

Most previous studies investigating the differentiation process of BMSCs used primary cells isolated from humans. BMSCs isolated from rat functions across barriers and could be used as a source of regenerative therapy for bone metabolic diseases and bone defects. van de Peppel et al. (2017) discriminated the first 4 days of human BMSCs differentiation into three distinct phases and presented candidate genes for early regulation of osteogenic and adipogenic lineage-commitment. In the present study, we focused on the mechanism underlying the determination of osteogenesis, adipogenesis, and chondrogenesis of rat BMSCs. Primary rat BMSCs represent a heterogeneous cell population, but by using inductive differentiation mediums, the clock of the cells was synchronized to limit cell-cycle heterogeneity. Three time points (3, 12, and 72 h) in three phases of early lineage-commitment of rat BMSCs differentiation were chosen and the high-throughput sequencing data we generated discovered the key transcription factors guiding the differentiation direction. These processes occurred before time windows examined by most previous studies but were proved to play critical roles in the differentiation (Ng et al., 2008; Piek et al., 2010). Hopx and other early responder TFs may control the osteogenic cell fate of BMSCs and participate in the development of osteoporosis. Gbx2 and other early responder TFs should be considered in mechanistic models that clarify cartilage-anabolic changes in the clinical progression of osteoarthritis.

In the first phase of differentiation, gene expression analyses revealed a high number of differentially expressed TFs in all three directions of differentiation. In the regulatory model derived from our data, the first phase represents the initiation stage of the differentiation program and is characterized by expression changes of many transcription-related genes that regulate lineage commitment and set the stage for further differentiation toward a stable phenotype. Downstream analyses of the differentially expressed TFs identified at 3 h of osteogenic differentiation showed that they were capable of regulating 92 and 46 DEGs in the second and third phases, respectively. As for the second phase, differentiating lineages begin to deviate, as reflected by the fact that many transcriptional changes are direct targets of the differentially expressed TFs in the first phases. In the current study, TFs that changed per hour decreased as time extended, which suggested that the differentiating cells had reached a stable phenotype. This illustrates the transition from proliferation to differentiation and reflects the inverse correlation between these processes as has been described in various other differentiating cells.

Interestingly, we identified two TFs (Tsc22d3 and Epas1) that were upregulated in three lineages at all three time points, which may play an important role in the initiation of osteogenesis, adipogenesis, and chondrogenesis. Tsc22d3, also known as glucocorticoid−induced leucine zipper, is a glucocorticoid−responsive anti−inflammatory molecule that could regulate intracellular signaling pathways via hypoxia-induced factor-1α (HIF−1α) as well as AP−1 (Kanda et al., 2020). Tsc22d3 is also one of the most regulated genes in Cushing’s syndrome (CS) and has been demonstrated to regulate osteoblast and bone turnover. Epas1, also known as HIF-2, is an active nucleoprotein and transcription factor induced by hypoxia. It could regulate hundreds of genes including VEGF, Ang-2, and HO-1, acting to enhance the production of corresponding proteins, and launch the process of angiogenesis and osteogenesis (Zhou et al., 2020). Thus, transfection of Epas1 into BMSCs provides an alternative for treating bone defects in the clinic. Other noteworthy TF is Hopx, which is the smallest member of the homeodomain-containing protein family (Hng et al., 2020). Hopx manifested an up-regulated transcription level only under the condition of osteogenesis (3, 12, and 72 h). Unlike other typical homeobox proteins, Hopx binds to different protein partners and therefore recruits transcription factors to gene promoter regions (Trivedi et al., 2010; Hng et al., 2020). Hemming et al. (2016) have demonstrated a correlation between the Enhancer of zeste homolog 2 (EZH2) and Hopx, implicating a regulatory effect of Hopx during BMSC osteogenesis. Similarly, Gbx2 may participate in the decision of chondrogenesis of BMSCs. This TF is a LIF/STAT3 signaling downstream target that could allow long-term expansion of the undifferentiated embryonic stem cells (Wang et al., 2017). Sox9 was up-regulated in chondrogenesis but down-regulated in adipogenesis in tree time points, which was consistent with previous studies that this TF was essential for lineage differentiation of BMSCs (Lefebvre et al., 2019). Cooperation between GLI, JUN/FOSL2, and Sox9 is based on multiple binding sites near those of Sox9 and therefore transactivates many cartilage-specific genes (Liu and Lefebvre, 2015). Four TFs including Ybx3, Zbtb25, Hes6, and Zfp879 were inclusively upregulated at 3 h of osteogenesis. Further studies are needed to elucidate their role in the initiation of osteogenesis.

Then we used a variety of bioinformatics analysis methods to analyze these differentially expressed TFs to obtain a comprehensive understanding. As a result, GO terms of the differentially expressed TFs at 3 h of osteogenesis, adipogenesis, and chondrogenesis for biological process categories included “negative regulation of transcription from RNA polymerase II promoter,” “transcription, DNA-templated,” and “regulation of transcription, DNA-templated.” Furthermore, the KEGG pathway enrichment analysis revealed that the differentially expressed TFs at 3 h of osteogenesis and adipogenesis were predominantly associated with the “signaling pathways regulating pluripotency of stem cells” (Id2, Id3, and Klf4). Id2 and Id3 were downregulated in three lineages while Klf4 was upregulated in the process of adipogenesis and osteogenesis. This suggests that the initiation of BMSCs differentiation is similarly activated in both lineages, and changes of these signaling pathways and TFs are necessary to exit the immature multi-potent cell stage and acquire a specialized mesenchymal phenotype. While in the second phase, the enriched KEGG pathways begin to deviate. For osteogenesis, “signaling pathways regulating pluripotency of stem cells” was still significantly enriched, while for adipogenesis, “PI3K-Akt signaling pathway” and “MAPK signaling pathway” were significantly enriched. For chondrogenesis, “cAMP signaling pathway” was significantly enriched.

The regulatory network provided potential connections between identified DEGs and TFs. There were four, six, and six differentially expressed TFs identified at 3 h during osteogenesis, adipogenesis, and chondrogenesis possessing targets among DEGs identified at all three time points. It could be noticed that Cenpa and Epas1 overlap in osteogenesis and adipogenesis while Sox9 overlaps in adipogenesis and chondrogenesis. Like Epas1, Cenpa was also upregulated in both osteogenesis and adipogenesis at 3 h. Cenpa is a functionally conserved molecule about 17kDa that forms a centromere-specific nucleosome with H2A, H2B, and H4, whose overexpression is crucial for multiple kinds of cancers (Saha et al., 2020; Zhang et al., 2020). The role of Cenpa in lineage-commitment of BMSCs has not to be raised by former studies.

It could be noticed that there are some variations at the transcriptional level between the differentiation of human and rat BMSCs. At present, mechanism investigation regarding the lineage-commitment of rat BMSCs is still lacking. We performed high-throughput transcriptome sequencing and conducted a rigorous informatics analysis based on multiple databases, proposing multiple key transcription factors that may participate in the lineage-commitment of rat BMSCs. These findings provide research directions and potential molecular candidates for further studies investigating the underlying mechanisms of three-directional differentiation of rat BMSCs. However, this study has some shortcomings. First, this is an *in vitro* experiment based on isolated rat BMSCs. The differentiation processes may possess different characteristics *in vivo*. Findings obtained from *in vitro* experiments cannot simply be transposed to predict the reaction of an entire organism *in vivo*. Second, only three time points were selected in this study. These time points were separately locating in three phases and were used as delegates. Third, further in-depth studies are needed to establish whether MSCs from adipose tissue or other anatomical locations undergo a similar cascade of transcriptional events. Adipose-derived MSCs may respond very differently with respect to their clonogenic potential and doubling time in comparison with BMSCs (Russo et al., 2014). Fourth, the detailed functional mechanism of identified TFs was not clearly elucidated, and we could not attribute the lineage-commitment to a particular TF (or set of TFs). Last but not least, because there was a large and diverse amount of data, we could not validate these TFs by western blotting. Proteomics study may be needed in the future.

## Conclusion

These transcription factors, functional terms and predicted targeted genes identified in the current study can help shed light on the molecular mechanism underlying the lineage-commitment of rat bone mesenchymal stem cells. Changes in TF activity during the early stage may play pivotal roles in the regulation of subsequent later phases of mesenchymal differentiation. Hopx and other early responder TFs may control the osteogenic cell fate of BMSCs, while Gbx2 and other early responder TFs should be considered in mechanistic models that clarify cartilage-anabolic changes. The latter finding exhibits possibilities for efficiently inducing osteoblast and chondrocytes differentiation, as part of a bone anabolic strategy for osteoporosis and osteoarthritis. In-depth experiments are required to further clarify the effect of these transcription factors.

## Data Availability Statement

The datasets presented in this study can be found in online repositories. The names of the repository/repositories and accession number(s) can be found below: https://www.ncbi.nlm.nih.gov/geo/query/acc.cgi?acc=GSE185140.

## Ethics Statement

The animal study was reviewed and approved by the Animal Ethics Committee of Shandong Provincial Hospital affiliated to Shandong First Medical University.

## Author Contributions

FL and QZ conceived and designed the study. FL, QZ, JD, and DZ analyzed RNA sequencing data. FL, QZ, and PZ performed the basic experiment. FL and QZ wrote the manuscript, which was commented on by all authors.

## Conflict of Interest

The authors declare that the research was conducted in the absence of any commercial or financial relationships that could be construed as a potential conflict of interest.

## Publisher’s Note

All claims expressed in this article are solely those of the authors and do not necessarily represent those of their affiliated organizations, or those of the publisher, the editors and the reviewers. Any product that may be evaluated in this article, or claim that may be made by its manufacturer, is not guaranteed or endorsed by the publisher.

## Footnotes

1
http://bioinfo.life.hust.edu.cn/AnimalTFDB/#!/

2
http://www.geneontology.org/

3
https://david.ncifcrf.gov/summary.jsp

4
http://string-db.org

## Supplementary Material

Supplementary Figure 1 Volcano plot and heatmap of differentially regulated TFs during adipogenesis at 3 h **(A,B)**, 12 h **(C,D)**, and 72 h **(E,F)** compared with undifferentiated cells (*t* = 0 h).

Supplementary Figure 2 Volcano plot and heatmap of differentially regulated TFs during chondrogenesis at 3 h **(A,B)**, 12 h **(C,D)**, and 72 h **(E,F)** compared with undifferentiated cells (*t* = 0 h).

Supplementary Figure 3 GO including biological process, cellular components and molecular function analysis, and KEGG enrichment analysis of differentially expressed TFs at 3 h **(A)**, 12 h **(B)**, and 72 h **(C)** of adipogenesis compared with undifferentiated cells (*t* = 0 h).

Supplementary Figure 4 GO including biological process, cellular components and molecular function analysis, and KEGG enrichment analysis of differentially expressed TFs at 3 h **(A)**, 12 h **(B)**, and 72 h **(C)** of chondrogenesis compared with undifferentiated cells (*t* = 0 h).

Supplementary Figure 5 Protein-protein interaction (PPI) network analysis of differentially expressed TFs at 3 h **(A)**, 12 h **(B)**, and 72 h **(C)** of osteogenesis, and 3 h **(D)**, 12 h **(E)**, and 72 h **(F)** of chondrogenesis compared with undifferentiated cells (*t* = 0 h). Red indicates upregulated TFs, and green indicates the downregulated TFs.

Supplementary Figure 6 The regulatory network between differentially expressed TFs and targeted differentially expressed genes at 12 and 72 h in osteogenesis **(A,B)**, adipogenesis **(C,D)**, and chondrogenesis **(E,F)** compared with undifferentiated cells (*t* = 0 h). Blue indicates TFs, and green indicates targeted genes.

Supplementary Figure 7 The regulatory network between differentially expressed TFs and targeted differentially expressed genes between every two lineages at 3 h after inductive differentiation (*t* = 0 h). **(A)** comparison between osteogenesis and adipogenesis; **(B)** comparison between osteogenesis and chondrogenesis, and **(C)** comparison between chondrogenesis and adipogenesis. Blue indicates TFs, and green indicates targeted genes.
