# Supplementary figures and images for: Dynamics of Transcription Factors in Three Early Phases of Osteogenic, Adipogenic, and Chondrogenic Differentiation Determining the Fate of Bone Marrow Mesenchymal Stem Cells in Rats
Source: Front Cell Dev Biol. 2021 Oct 26;9:768316. doi: 10.3389/fcell.2021.768316 (PMC8576568; doi:10.3389/fcell.2021.768316)

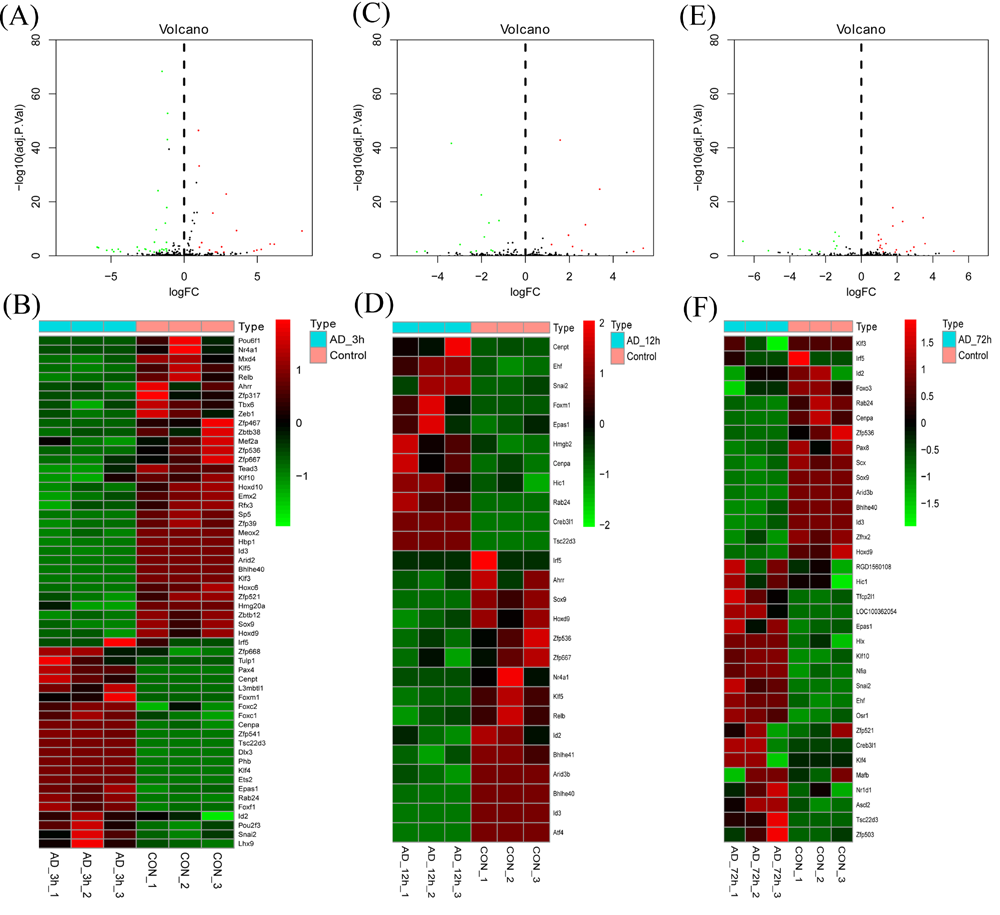

Supplement: Supplementary Figure 1 — Volcano plot and heatmap of differentially regulated TFs during adipogenesis at 3 h (A,B), 12 h (C,D), and 72 h (E,F) compared with undifferentiated cells (t = 0 h). [file Image_1.TIFF]

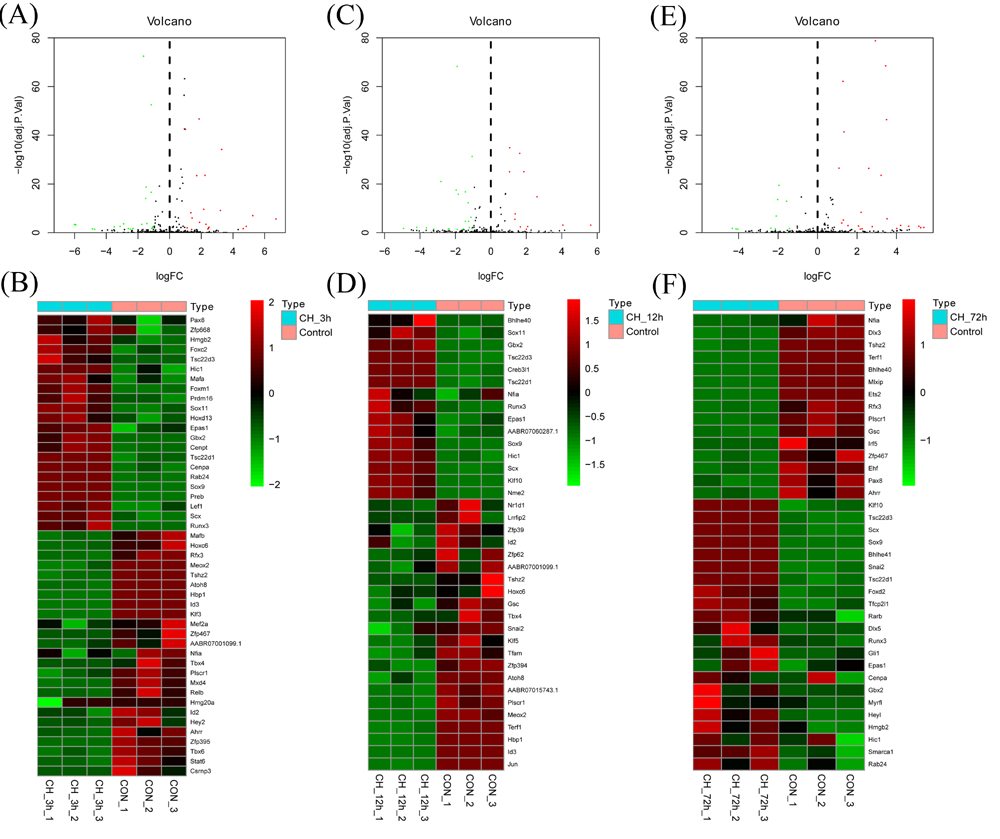

Supplement: Supplementary Figure 2 — Volcano plot and heatmap of differentially regulated TFs during chondrogenesis at 3 h (A,B), 12 h (C,D), and 72 h (E,F) compared with undifferentiated cells (t = 0 h). [file Image_2.TIFF]

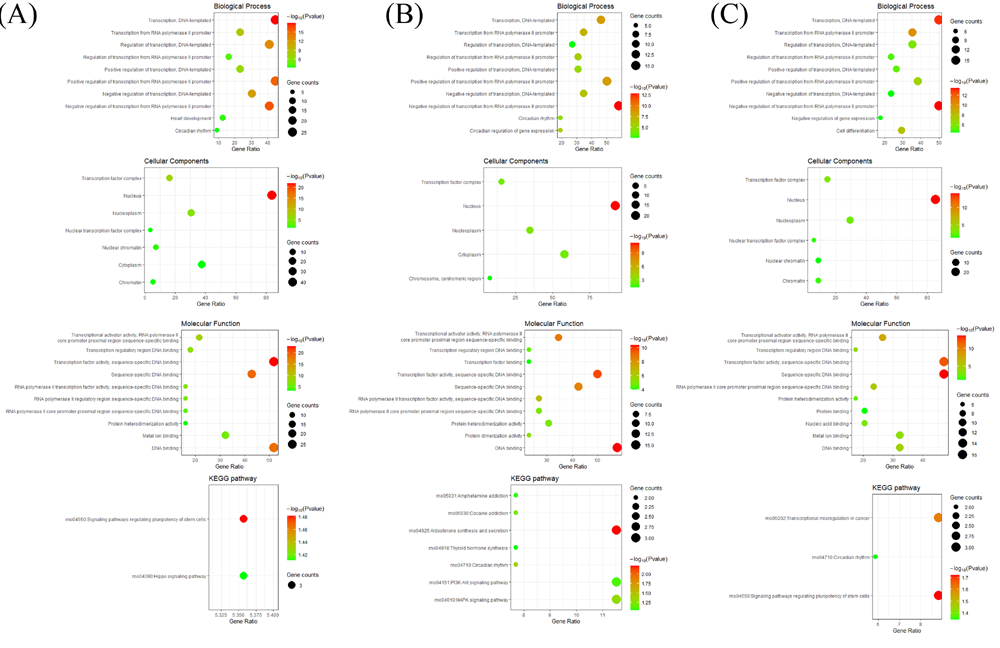

Supplement: Supplementary Figure 3 — GO including biological process, cellular components and molecular function analysis, and KEGG enrichment analysis of differentially expressed TFs at 3 h (A), 12 h (B), and 72 h (C) of adipogenesis compared with undifferentiated cells (t = 0 h). [file Image_3.TIFF]

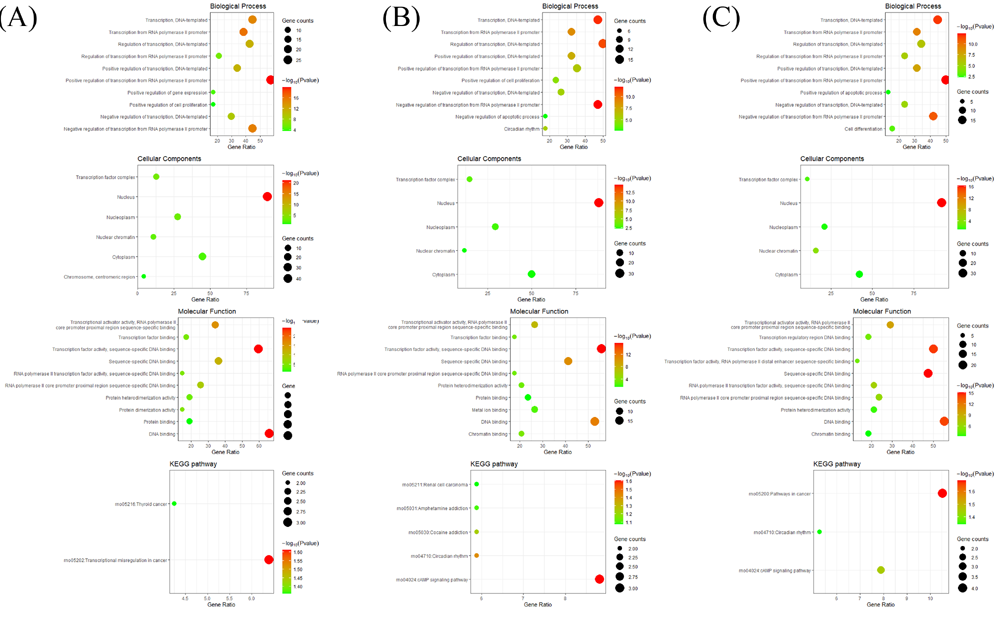

Supplement: Supplementary Figure 4 — GO including biological process, cellular components and molecular function analysis, and KEGG enrichment analysis of differentially expressed TFs at 3 h (A), 12 h (B), and 72 h (C) of chondrogenesis compared with undifferentiated cells (t = 0 h). [file Image_4.TIFF]

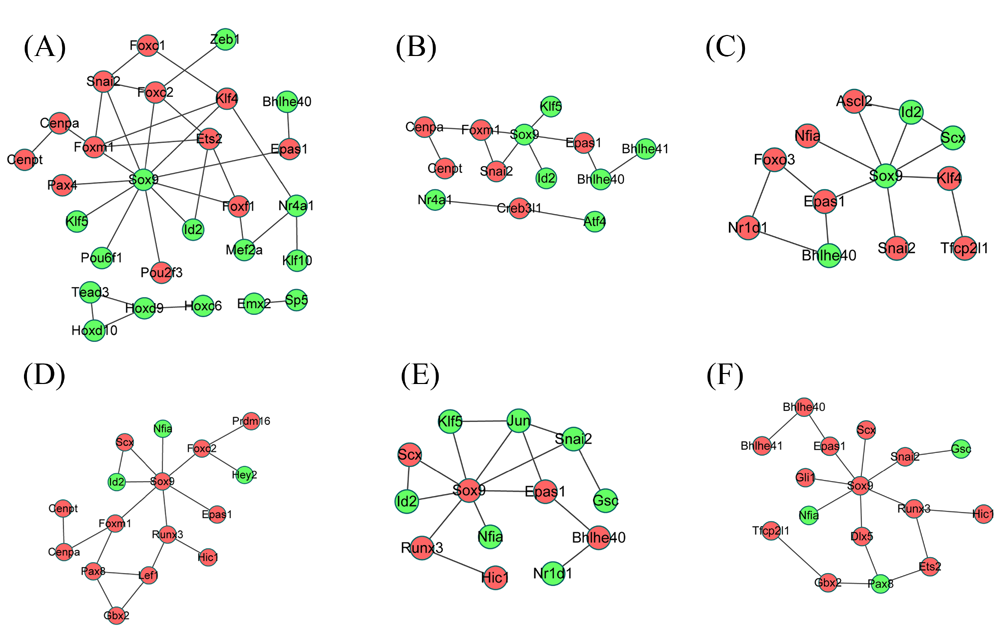

Supplement: Supplementary Figure 5 — Protein-protein interaction (PPI) network analysis of differentially expressed TFs at 3 h (A), 12 h (B), and 72 h (C) of osteogenesis, and 3 h (D), 12 h (E), and 72 h (F) of chondrogenesis compared with undifferentiated cells (t = 0 h). Red indicates upregulated TFs, and green indicates the downregulated TFs. [file Image_5.TIFF]

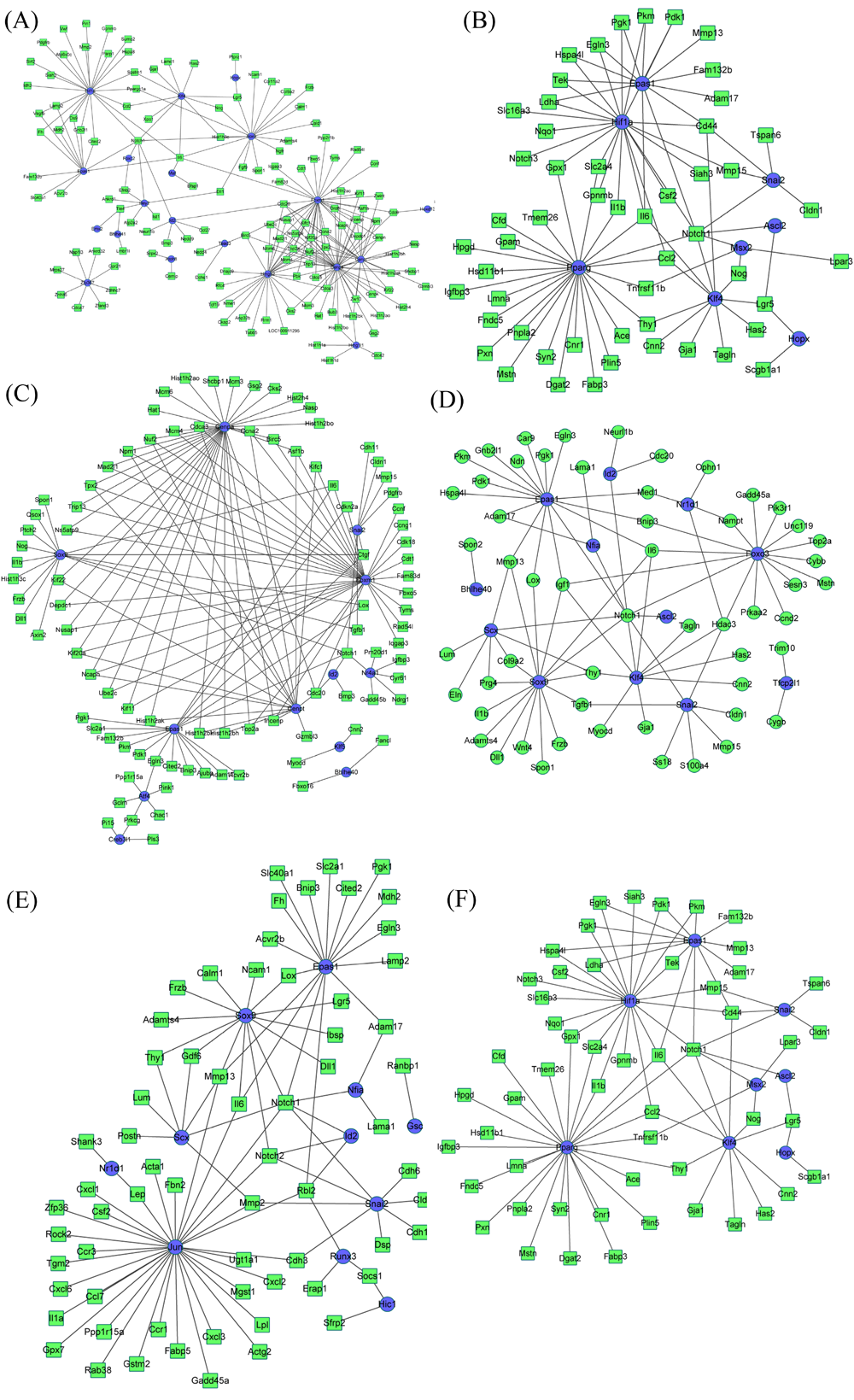

Supplement: Supplementary Figure 6 — The regulatory network between differentially expressed TFs and targeted differentially expressed genes at 12 and 72 h in osteogenesis (A,B), adipogenesis (C,D), and chondrogenesis (E,F) compared with undifferentiated cells (t = 0 h). Blue indicates TFs, and green indicates targeted genes. [file Image_6.TIFF]

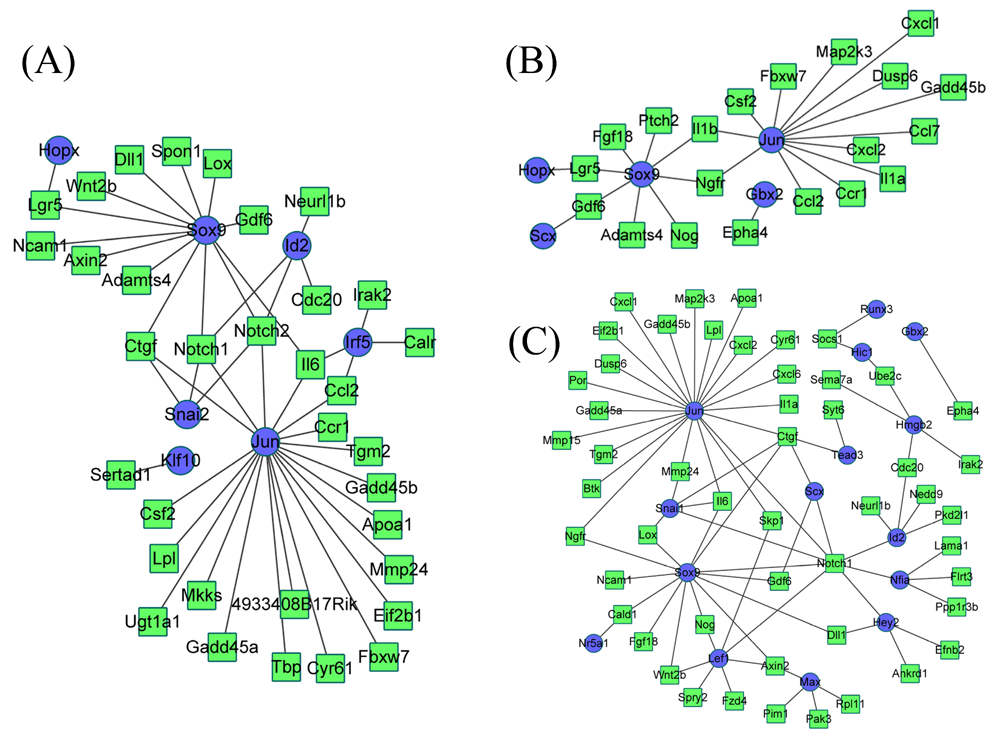

Supplement: Supplementary Figure 7 — The regulatory network between differentially expressed TFs and targeted differentially expressed genes between every two lineages at 3 h after inductive differentiation (t = 0 h). (A) comparison between osteogenesis and adipogenesis; (B) comparison between osteogenesis and chondrogenesis, and (C) comparison between chondrogenesis and adipogenesis. Blue indicates TFs, and green indicates targeted genes. [file Image_7.TIFF]
